# Lack of Evidence for Zoonotic Transmission of Schmallenberg Virus

**DOI:** 10.3201/eid1811.120650

**Published:** 2012-11

**Authors:** Chantal Reusken, Cees van den Wijngaard, Paul van Beek, Martin Beer, Ruth Bouwstra, Gert-Jan Godeke, Leslie Isken, Hans van den Kerkhof, Wilfrid van Pelt, Wim van der Poel, Johan Reimerink, Peter Schielen, Jonas Schmidt-Chanasit, Piet Vellema, Ankje de Vries, Inge Wouters, Marion Koopmans

**Affiliations:** National Institute for Public Health and the Environment, Bilthoven, the Netherlands (C. Reusken, C. van den Wijngaard, P. van Beek, G.-J. Godeke, L. Isken, H. van den Kerkhof, W. van Pelt, J. Reimerink, P. Schielen, A. de Vries, M. Koopmans);; Friedrich-Loeffler-Institut, Insel Riems, Germany (M. Beer);; Central Veterinary Institute of Wageningen University and Research Centre, Lelystad, the Netherlands (R. Bouwstra, W. van der Poel);; Bernhard Nocht Institute for Tropical Medicine, Hamburg (J. Schmidt-Chanasit);; Animal Health Service, Deventer, the Netherlands (P. Vellema);; and Institute for Risk Assessment Sciences, Utrecht, the Netherlands (I. Wouters)

**Keywords:** Bunyaviridae, emerging infection, arbovirus, Schmallenberg virus, zoonoses, public health, transmission, arthropod-borne viruses, the Netherlands, Europe

## Abstract

The emergence of Schmallenberg virus (SBV), a novel orthobunyavirus, in ruminants in Europe triggered a joint veterinary and public health response to address the possible consequences to human health. Use of a risk profiling algorithm enabled the conclusion that the risk for zoonotic transmission of SBV could not be excluded completely. Self-reported health problems were monitored, and a serologic study was initiated among persons living and/or working on SBV-affected farms. In the study set-up, we addressed the vector and direct transmission routes for putative zoonotic transfer. In total, 69 sheep farms, 4 goat farms, and 50 cattle farms were included. No evidence for SBV-neutralizing antibodies was found in serum of 301 participants. The lack of evidence for zoonotic transmission from either syndromic illness monitoring or serologic testing of presumably highly exposed persons suggests that the public health risk for SBV, given the current situation, is absent or extremely low.

## Abstract 2

The risk to public health is absent or extremely low.

In November 2011, scientists in Germany identified novel viral sequences in serum from cattle affected by a febrile syndrome that was reported during August–September 2011 in Germany and the Netherlands. Clinical signs included decreased milk production and diarrhea. The virus, named Schmallenberg virus (SBV), was isolated from blood of affected cattle, and similar clinical manifestations were observed in experimentally infected calves (*1*). In the Netherlands, SBV was detected retrospectively in serum from affected cattle in December 2011 (*2*).

Since the end of November 2011, an unusually high number of ovine and bovine congenital malformations were reported in the Netherlands. The main macroscopic findings included arthrogryposis; torticollis; scoliosis; brachygnathia inferior; hydranencephaly; and hypoplasia of cerebrum, cerebellum, and spinal cord. SBV genome was detected in the brain of malformed lambs and calves (*3*–*5*). These findings, together with detection of SBV RNA in multiple types of samples, e.g., amniotic fluid, meconium, and placenta remains from diseased lambs and calves, strongly pointed to SBV as the causative agent of the clinical manifestations (*6*). The teratogenic effects in ruminants are hypothesized to reflect virus circulation in late summer/early autumn 2011, leading to intrauterine infection with SBV during a specific period of gestation (*4*).

In June 2012, seven additional European countries (Belgium, Denmark, France, Italy, Luxemburg, Spain, and the United Kingdom) confirmed SBV in ruminants, accumulating to a total of 3,745 PCR-confirmed infected animal holdings (*4*,*7*). In the Netherlands 1,670 holdings were suspected to be affected by SBV on the basis of births of animals with malformations typical of SBV infection, of which 350 were confirmed by PCR as of June 12, 2012. The holdings with confirmed SBV comprise 237 cattle, 107 sheep, and 6 goat farms (*8*).

SBV has been identified as most related to Sathuperi virus, and for the small and large segments, Shamonda virus segments show the highest sequence identity. All those viruses are members of the Simbu serogroup, family *Bunyaviridae*, genus *Orthobunyavirus*, and known as arthropod-borne viruses that can cause illness in ruminants (*9*). The orthobunyaviruses comprise ≈170 virus isolates, assigned to 48 distinct species, arranged in 18 serogroups, including the Simbu serogroup. Serogroups within the genus are based on cross–hemagglutination-inhibition and antibody neutralization relationships. Phylogenetic relationships are consistent with the results of serologic relationships (*10*–*12*).

Because the family *Bunyaviridae* contains several medically relevant zoonotic viruses, of which Crimean-Congo hemorrhagic fever virus, Rift Valley fever virus, Sin Nombre virus, and sandfly fever Naples virus are examples, the emergence of SBV triggered a joint veterinary and public health response in the Netherlands to address the possible consequences to human health. We present the public health risk ascertainment of the emergence of SBV in ruminants in the Netherlands and most likely other European countries were SBV has emerged.

## Methods

### Profiling Risks to Humans

We used a standard in-house checklist for profiling the risk to human health of novel emerging viruses to assess the public health risks for SBV. This checklist comprised 10 items: 1) situation assessment; 2) review of taxonomic position of the newly identified virus; 3) review of human health risks associated with closely related viruses; 4) review of epidemiology of related viruses (transmission cycle, reservoirs, and vectors); 5) review of clinical manifestations in humans of related viruses (including kinetics of immune response and shedding); 6) assessment of potential for human exposure and identification of related risk factors; 7) assessment of human diagnostics; 8) design of a literature/evidence-based testing algorithm; and 10) conclusions and recommendations.

### Virus and Validation Serum

An SBV strain, isolated from SBV reverse transcription PCR–positive, homogenized brain tissue of a malformed lamb in the Netherlands, was obtained from the Central Veterinary Institute (Lelystad, the Netherlands). Putative cross-reacting orthobunyaviruses circulating in Europe, Batai virus (*13*), Tahyna virus (*14*), and Inkoo virus (*15*), were obtained from the Bernhard Nocht Institute for Tropical Medicine (Hamburg, Germany). All viruses were propagated and titrated (50% tissue culture infectious dose [TCID_50_]) in continuous African green monkey kidney cells (Vero E6, ATCC CRL-1586). SBV-positive control serum from a ewe that had given birth to an SBV PCR-positive lamb was obtained from the Animal Health Service (AHS), and positive serum sample from an experimentally infected ewe was obtained from the Central Veterinary Institute.

Well-defined negative and positive human serum cohorts were not available because SBV is a novel emerging virus with unknown zoonotic potential. Therefore, we validated the virus neutralization test (VNT) using presumed seronegative serum from 1) 56 patients without travel history submitted to the National Institute for Public Health and the Environment during February 28, 2007–February 25, 2008, for routine diagnostic testing for *Bordetella pertussis*; 2) 73 inhabitants of municipalities with known SBV activity in 2011 that had been collected during August 15, 2010–October 15, 2010, for routine screening; and 3) 93 veterinary students collected in 2006 and 2008. Serum from 92 veterinary students sampled during 2011 and from 73 inhabitants of municipalities with known SBV activity collected during August 15, 2011–October 15, 2011, for routine screening were considered to represent community samples from possibly exposed populations and were added to the validation panel. Anonymized use of serum from the National Institute for Public Health and the Environment was covered by the rules of the code of conduct for proper use of human tissue of the Dutch Federation of Medical Scientific Associations. The cohort study of the veterinary students included screening for zoonotic infections and was approved by the Medical Ethical Committee of the University Medical Centre Utrecht.

### VNT

For VNT, Vero E6 cells were seeded in 96-well plates and incubated overnight at 37°C with 5% CO_2_ until the cells were ≈80%–90% confluent. Serum was heated for 30 min at 56°C to inactivate complement before use. Serum was serially diluted in 2-fold steps in minimum essential medium (GIBCO/Life Technologies, Bleiswijk, the Netherlands). We added 100 TCID_50_ of virus to the diluted serum (volume of 60 µL each). To rule out the presence of other cytopathic effect–inducing factors, serum dilutions also were added to control wells to which no virus was added. After incubation at 37°C in 5% CO_2_ for 1 h, 100 µL of the virus-plus-serum mixture, no virus-serum controls, and a virus dilution control were added to the Vero E6 cells and incubated for 3 d at 37°C. Assays were performed in duplicate. Cells were monitored for cytopathic effect after 3 days.

### Monitoring of Health Symptoms

Persons in close contact with affected animals or their birth materials in whos fever developed (>38°C) within 2 weeks after exposure were asked to contact the regional public health service (PHS) for evaluation and assessment of the need for follow-up. This request was made through an email-based alert system hosted by the AHS and farmers association to veterinarians. The alert system prompted veterinarians to inform farmers on SBV-affected holdings. When a relation between reported fever and SBV was considered possible, a short questionnaire was filled in by study participants, and serum was tested by real-time PCR (as described in [*6*]) and VNT to diagnose a possible SBV infection.

### Design of Serologic Study in Persons with High Probability of Exposure

A serologic survey was designed to determine the presence of SBV antibodies in serum from persons living and working on farms where SBV had been highly suspected on the basis of pathologic findings consistent with typical SBV-induced malformations in calves or lambs, most confirmed by PCR and/or serology. The target cohort, consisting of adult (>18 years of age) farmers, farm residents, farm employees, and veterinarians who had been exposed to affected herds, were invited to participate by donating a serum sample and filling in a questionnaire. A total of 240 affected animal holdings were approached through direct mailing by the AHS. Employees of the regional PHS visited the affected farms and collected serum samples and questionnaires. The veterinarians were collectively contacted to be sampled at a national conference after a preannouncement of the purpose of the study.

The questionnaire addressed demographics, the animal species involved, the type and level of exposure (birth materials, feces, milk or other products, insects), protective equipment used during work, general health, (recent) health complaints, and presence of wounds on hands. The study protocol, information material, and questionnaires were assessed by the Medical Ethical Committee of the University Medical Centre Utrecht and approved (METC no. 12–106).

On the basis of a literature review of seroprevalence studies in regions with known orthobunyavirus outbreaks, a seroprevalence of 2% was established as the lower bound in an affected human population (N. Cleton, unpub. data; *16**–**19*). In this scenario with 2% seroprevalence, testing of, for example, 200 exposed persons would give a probability of 98.24% to detect >1 seropositive persons (Table 1).

**Table 1:** Probability of detecting at least 1 seropositive sample among different sample sizes and hypothetical seroprevalences in study to determine whether Schmallenberg virus can be zoonotically transmitted, the Netherlands

Sample size	Hypothetical seroprevalence, %	Probability* of detecting at least 1 seropositive, %
50	2.00	63.58
100	2.00	86.74
150	2.00	95.17
192	2.00	97.93
200	2.00	98.24
301	2.00	99.77
301	1.00	95.14
301	0.50	77.88
301	0.25	52.93
301	3.00	99.99

*The probability was calculated as 1 – (1 – seroprevalence) × sample size, so for a seroprevalence of 2% and a sample size of 200 the probability of detecting at least 1 seropositive = 1 – (1 – 0.02) × 200.

## Results

### Profiling the Human Risks for SBV

#### Human Disease in Related Viruses

The literature indicates that zoonotic transmission of SBV could not be completely ruled out. The taxonomic position of SBV had been identified as family *Bunyaviridae*, genus *Orthobunyaviru*s, Simbu serogroup (*1*). At least 30 orthobunyaviruses have been associated with human disease. Virologic or serologic evidence for zoonotic infection has been found for several viruses within the Simbu serogroup, including viruses considered to be primarily livestock pathogens (Aino and Shuni virus; Table 2). Among the many reasons for vigilance was the lack of full characterization of SBV. Genetic reassortment between orthobunyaviruses within the same serogroups has led to emergence of new viruses, occasionally with increased pathogenicity and potentially with changes in host range (*21*,*36*–*40*).

**Table 2:** Evidence for zoonotic infection within the family *Bunyaviridae*, *Orthobunyavirus* genus, the Netherlands*

Serogroup, species	Geographic distribution	Reservoir	Human infection	Evidence	Reported symptoms in humans	Congenital disease in humans	References
Simbu							
Oropouche virus	South America	Humans, sloths, marmosets	Yes	Outbreaks	Febrile illness, arthralgia, diarrhea	NR	(*18*,*20*)
Iquitos virus	South America	Humans, unknown	Yes	Unexplained fever surveillance	Febrile illness, arthralgia, diarrhea	NR	(*21*)
Akabane virus	Asia, Israel, Kenya, Australia	Cattle, horses, sheep	No	No serology	NR	NR	(*22*,*23*)
Shamonda virus	Africa	Cattle	No	No serology	NR	NR	(*24*,*25*)
Aino virus	Asia	Cattle	Possibly	Serology	Unknown	Unknown	(*22*,*23*,*26*)
Shuni virus	Africa	Cattle, horses, sheep	Possibly	Virus isolation (one case)	Fever, no hospital submission	NR	(*27*–*29*)
Sathuperi virus	Asia, Africa	Cattle	Unknown	Unknown	Unknown	Unknown	(*30*)
California encephalitis							
California encephalitis virus	North America	Rodents, lagomorphs	Yes	Endemic, common	Febrile illness, encephalitis	NR	(*31*)
La Crosse virus	North America	Rodents, lagomorphs	Yes	Endemic common	Febrile illness, encephalitis	NR	(*31*)
Tahyna virus	Europe, Asia, Africa	Lagomorphs, rodents, hedgehogs	Yes	Serology, cases	Febrile illness, respiratory symptoms meningitis (mild)	NR	(*32*)
Inkoo virus	Northern Europe	Lagomorphs	Yes	Surveillance febrile illness, CNS illness,	Febrile illness, Meningitis (mild)	NR	(*32*)
Snowshoe hare virus	North America, Far-Eastern Europe	Lagomorphs, rodents	Yes	Serology, case reports	Febrile illness	NR	(*32*) (*31*)
Bunyamwera							
Batai virus	Europe, Asia, Africa	Birds,pigs, horses, ruminants	Yes	Serology (rare)	Febrile illness, affection	NR	(*32*)
Cache Valley virus	North America	Deer, sheep, horses, cattle	Yes	2 Case reports, serology	Febrile illness, encephalitis	Under discussion	(*33*–*35*)
Ngari virus	Africa	Humans, unknown	Yes	Outbreaks, virus isolation	Febrile illness, hemorragic fever	NR	(*36*–*38*)

*NR, not reported; CNS, central nervous system.

#### Modes of Transmission

The related Shamonda, Sathuperi, Aino, and Akabane viruses are transmitted mainly by biting midges (*23*; *41* in Technical Appendix), and the epidemiology of the infection in animals and the first detections of SBV genome in *Culicoides* spp. midges in Belgium, Denmark, and Italy suggested vector-borne spread as a mode of transmission for SBV as well (*1**,**2*; *42*–*44* in Technical Appendix). In addition, the birth defects in lambs and calves increased the need for assistance from veterinarians during parturition, and high loads of viral RNA were detected in birth materials of sheep and cattle (*6*). Therefore, if SBV is zoonotic, transmission could have occurred to persons who could have been exposed to infected vectors (residents, farmers, veterinarians) and/or through direct contact with animals that had congenital malformations or with birth material, e.g., during assistance at deliveries (farmers, veterinarians). A testing algorithm was designed (Figure). Professionals were advised to respect common hygiene measures for veterinarian-assisted deliveries and handling of affected newborn ruminants. Pregnant women were advised not to assist at ruminant deliveries.

**Figure:**
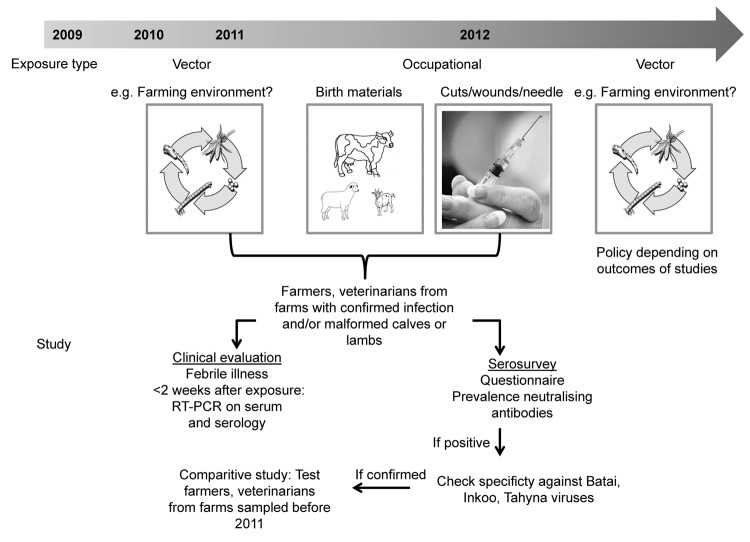
Testing algorithm to determine whether Schmallenberg virus can be zoonotically transmitted, the Netherlands. RT-PCR, reverse transcription PCR.

### Validation of VNT

Because the viremic phase in orthobunyavirus infections typically is short, we chose to use serologic testing by VNT to evaluate an immunologic response in exposed persons (*1*,*21*). For assay validation, possible cross-reacting zoonotic viruses circulating in Europe were identified. Zoonotic viruses in the Simbu serogroup are not known to circulate in Europe, but related orthobunyaviruses that may infect humans are Batai virus (BATV), Tahyna virus (TAHV), and Inkoo virus (INKV) (Table 2). No cross-neutralization was observed when the SBV-positive control serum was tested against 100 TCID_50_ of BATV, INKV, and TAHV, whereas the homologous titer was 512 (data not shown). The reverse experiment could not be conducted because of a lack of reference reagents. A control cohort of 222 serum samples, presumed negative on the basis of collection data before 2011, were all negative in the VNT (data not shown). Another validation cohort of 165 serum samples, possibly positive on the basis of collection data in 2011 and putative exposure through residence and professional activities, were all negative as well (data not shown).

### Monitoring of Symptoms

Symptoms that could be attributed to a putative infection with SBV were determined on the basis of an inventory made of syndromes related to human infection with closely related viruses of the Simbu group, i.e., Oropouche virus and Iquitos virus (Table 2). These viruses typically cause a febrile illness accompanied by chills, general malaise, headache, anorexia, muscle and joint pain, muscle weakness, and vomiting. Symptoms of meningitis or a rash occasionally develop. The reported diseases generally are self-limiting (*20*,*21*).

Because the range of symptoms described was diverse, we decided to monitor patients who suited our case definition: febrile disease >38°C within 2 weeks after contact with malformed calves or lambs or their birthing materials (in the absence of the supposed vector during the winter season). The 2-week period was based on the known incubation period for Oropouche virus in humans, typically 4–8 days (*20*). Eight cases were reported by the PHS during January 1–April 15, 2012. Four of these were excluded because they did not meet the case definition. The remaining 4 cases were tested by PCR and VNT (for 3 cases only because only vesicle fluid was available for 1 study participant). None of the tested suspected case-persons showed evidence of an SBV infection.

In addition, no unusual trends were noted during or since summer 2011 in the existing routine surveillances for neurologic illness, gastroenteritis, and influenza-like illness at the Netherlands Centre for Infectious Disease Control (H. van der Avoort, E. Duizer, and A. Meijer, pers. comm.).

### Serology in High-Exposure Groups

To enable evidence-based risk profiling, serologic surveillance was initiated in persons residing at locations with proven SBV circulation and professionals in close contact with infected animals and their birth materials. In this study set-up, we addressed the vector and the direct transmission routes for putative zoonotic transfer.

The study comprised 301 participants. Of these, 192 worked or lived on farms with laboratory-confirmed SBV circulation in animals, 42 persons worked or lived on farms where animals were being raised and where SBV infection was highly suspected, and 67 were veterinarians who had been in contact with malformed animals (Table 3, Table 4). These 123 farms consisted of 69 sheep, 4 goat, and 50 cattle farms that had animals with typical SBV malformations (no other pathogens were circulating in the Netherlands that cause congenital malformations, including arthrogryposis), of which most were PCR and/or VNT confirmed (83%; Table 4). SBV-specific antibodies were detected in livestock serum at 97.7% (83/85) of the farms for which serum was available (Table 4). Overall, 229 participants specifically reported direct exposure to newborn calves, lambs, and/or birth materials from SBV-infected herds; these participants comprised 179 farmers, and 50 veterinarians (39 of whom were exposed while assisting with deliveries at farms and 11 during postmortem examination of malformed newborns at the AHS). A total of 150 participants reported insect bites on SBV-infected farm(s), exposing them potentially to SBV during the vector season (Table 3).

**Table 3:** Main characteristics of study participants* in study to determine whether SBV can be zoonotically transmitted, the Netherlands*

Exposure group, risk factor	Exposed	Participants, no. (%)/additional information
All		
Total		301/age 18–88 y; mean 47 y; 25th–75th percentile 36–58 y; 62% male
Exposure to biting insects on SBV-infected farm(s)	Yes	150 (50)
	No	71 (24)
	Unknown	80 (26)
Farmers, total		234
Working and living on SBV-infected farm		191 (82)
Working on SBV-infected farm		26 (11)
Living on SBV-infected farm		15 (6)
Unknown		2 (1)
Exposure to animals		
Sheep		
Regular contact with lambs and/or birth products on SBV-infected farm	Yes	110, of whom 88 reported hand (skin) injuries during work
	No	31
Total		141
Goats		
Regular contact with kids and/or birth products on SBV-infected farm	Yes	3, of whom 3 reported regular hand (skin) injuries during work
	No	10
Total		13
Cattle		
Regular contact with calves and/or birth products on SBV-infected farm	Yes	90, of whom 72 reported regular (hand) skin injuries during work
	No	38
Total		128
Veterinarians		
Total		67/1–50 SBV-infected farms visited per veterinarian; median 4
Exposure to animals		
Sheep		
Contact with malformed lambs and/or birth products	Yes†	19, of whom 18 reported regular hand (skin) injuries during work
	No	29
Total		48
Goats		
Contact with malformed lambs and/or birth products	Yes†	1 who reported regular hand (skin) injuries during work
	No	29
Total		30
Cattle		
Contact with malformed calves and/or birth products	Yes†	33, of whom 28 reported regular hand (skin) injuries during work
	No	20
Total		53
Contact with malformed lambs/calves during section at Animal Health Service	Yes	11, of whom 6 reported regular hand (skin) injuries during work.

*Overall, 50 (75%) of 67 veterinarians reported contact with malformed lambs/calves and/or birth products of which 40 reported regular hand (skin) injuries during work. Overall, 179 (76%) of 234 farmers, farm residents and farm employees reported regular contact with newborn lambs/calves and/or birth products on SBV-infected farms, of which 140 reported regular hand (skin) injuries during work. SBV, Schmallenburg virus. †All tested seronegative.

**Table 4:** Characteristics of participating farms and number of human participants in study to determine whether SBV can be zoonotically transmitted, the Netherlands*

Animal species†	No. farms (no. animals/farm) [median])	Laboratory-confirmed SBV infection in animals, no. (%)				No. human participants	
		PCR	VNT	PCR and/or VNT		Total (no. per farm)	No. (%) from farms with laboratory-confirmed SBV infection
Sheep	69 (5–1,676 [83])	48 (70)	44 (64)	61 (88)		130 (1–6)	112 (86)
Goat	4 (4–1144 [759])	1 (25)	2 (50)	2 (50)		8 (1–4)	2 (25)
Cattle	50 (23–468 [136])	4 (8)	37 (74)	39 (78)		96 (1–5)	78 (81)
Total	123	53 (43)‡	83 (68)§	102 (83)		234	192 (82)

*A total of 240 farms were invited to participate in the study (113 sheep, 7 goat, and 120 cattle farms; all were highly suspected to be SBV infected on the basis of pathologic findings consistent with typical malformations in calves or lambs). 123 (51%) farms responded. SBV, Schmallenburg virus; VNT, virus neutralization test. †Mixed farms were classified according to the main animal species. ‡For 9 farms, PCR results not available. §For 38 farms, VNT results not available.

None of the 301 participants showed serologic evidence of SBV infection in the VNT, whereas a titer of neutralizing antibodies was high in the ovine control serum. In a scenario of 2% seroprevalence, testing of 301 persons would have led to a probability of 99.77% to detect >1 seropositive persons (97.93% on the basis of 192 persons with laboratory-confirmed exposure; Table 1). Nevertheless, sporadic infections cannot be excluded entirely.

## Discussion

The Netherlands has an integrated structure for human–animal risk analysis and response to zoonoses, established after the massive Q fever outbreak in 2007–2010. The continuous emergence of zoonotic viruses from livestock reservoirs, with examples of Nipah virus, Japanese encephalitis virus, highly pathogenic avian influenza A (H7N7) and A (H5N1) viruses, and coronaviruses, underscores the relevance of the One Health approach in assessing the risks for novel emerging pathogens, such as SBV (*45*–*49* in Technical Appendix). The emergence of SBV in 2011 was a test case for this collaborative approach to risk assessment. Information, protocols, and samples were shared rapidly, facilitating a quick public health response.

On the basis of the findings of an in-house risk-assessment algorithm, we concluded that zoonotic transmission of the virus could not be excluded, triggering the study described here. We found no evidence for infection by serology, but ruling out zoonotic infections with high certainty is not simple, particularly in a complex situation with >1 possible mode of transmission.

If zoonotic, transmission of SBV could have occurred through vector-borne transmission during the period of high vector density in summer and fall 2011. The level of exposure to SBV by arthropods depends on the vector capacity of the residing vectors. Vector capacity is a measure of the efficiency of vector-borne disease transmission comprising vector competence, susceptible host density, vector host feeding preferences, vector survival rate, vector density, and vector feeding rates (*50* in Technical Appendix). In this study, we found no evidence for human SBV infection, despite the high infection rate of sheep and cattle in the same localities (up to 100% within-herd seroprevalence (*51* in Technical Appendix) and the high level of reported insect bites during work on SBV-infected farms. From the high infection rates in ruminants, we conclude that the capacity of residing vectors to transmit SBV to cattle and sheep was high, indicating that vector-competence, vector densities, and vector survival rates were sufficient for SBV transmission. Therefore, the absence of SBV antibodies in humans implies that humans are not susceptible to SBV infection but only under the assumption that the vectors of SBV have host feeding preference for humans. Research into the host preferences of identified SBV vector species and, if proven anthropophilic, their feeding rates could clarify this issue.

If vector transmission would have been a route for zoonotic transmission leading to 2% seroprevalence in exposed persons, i.e., persons reporting insect bites on SBV-infected farms, in this study the probability of detecting at least 1 of such seropositive persons would have been 99.77%. However, this calculation is based on an assumed test specificity and sensitivity of 100%. A high specificity was justified on the basis of the negative results with the 387 control serum and the absence of neutralizing capacity of an SBV-positive ovine serum sample to INKV, BATV, and TAHV. Because SBV is a novel pathogen, no well-defined seropositive human serum cohorts were available to assay the analytical sensitivity of our test. However, even with sensitivity as low as 90%, the probability of detecting at least 1 seropositive person still would have been 99.69% (data not shown).

The second possible exposure could occur through contact with affected animals and/or birth materials. The congenital malformations in lambs and calves with SBV infection are such that increased assistance during delivery was needed from farmers and veterinarians. Direct exposure to newborn ruminants and/or birth materials was reported in 76% of the study participants. If contact during delivery would have been an active route for zoonotic transmission, leading to 2% seroprevalence in exposed persons, the probability of detecting at least 1 of such seropositive persons would have been 99.02%.

A third option is that exposure to newborns and their birth materials has a higher risk for infection if exposed persons had blood contact with the affected materials (e.g., by hand wounds). Sixty percent of participants reported small wounds on hands; thus, the probability of detecting such seropositives would have been high (i.e., 97.37% with 2% seroprevalence). In addition, 2 persons in the syndromic monitoring reported needlestick incidents, again without any evidence for infection through antibody testing.

The absence of evidence for direct transmission of SBV from ruminants to humans is in line with observations for other Simbu serogroup viruses (Akabane and Shamonda) infecting livestock (Table 2). Moreover, a serologic survey of 60 sheep farmers with sheep husbandry in the SBV epizootic area in Germany yielded no evidence for human SBV infection. However, of these farmers, only 48 had contact with lambs with SBV characteristic malformations, whereas SBV was laboratory confirmed in the livestock of only 36 participants (*52* in Technical Appendix), but the level of exposure through contact with affected animals and/or birth material is difficult to quantify (*4*). In the Netherlands, SBV RNA has been detected in the brains of malformed animals on 18.6% of reported cattle farms and on 30.6% of reported sheep farms (*8*), and high loads of viral RNA have been detected in some placentas and in birth fluids.

Current data suggest that infections might have been cleared by the time of delivery, particularly in cattle, which have longer gestations. Furthermore, finding RNA in birth materials does not give any information about the actual presence of infectious virus particles in these materials. Attempts to isolate viruses from such specimens have met with little success, and further research is needed to address the issue of infectivity of birth materials. This lack of virus isolation implies that the number of persons in this study directly exposed to infectious virus particles from affected animals and/or birth material might be lower than assumed on the basis of the number of participants reporting this exposure. Nevertheless, the lack of seropositive samples indicates that the risk for infection through contact with contaminated materials, regardless of whether they contain infectious virus particles, is minimal. Therefore, given the high seroprevalence of SBV in affected herds (*51* in Technical Appendix), the lack of any evidence for zoonotic transmission from either the syndromic monitoring or this serologic study suggests that the public health risk for SBV given the current situation is absent or extremely low.

Technical Appendix Supplementary references *41–52*.
