# Supplementary material for: Lack of Evidence for Zoonotic Transmission of Schmallenberg Virus
Source: Emerg Infect Dis. 2012 Nov;18(11):1746–54. doi: 10.3201/eid1811.120650 (PMC3559138; doi:10.3201/eid1811.120650)
Supplement: Technical Appendix — Supplementary references 41–52. [file 12-0650-Techapp-s1.pdf]

Article DOI: http://dx.doi.org/10.3201/eid1811.120650

Lack of Evidence for Zoonotic Transmission of Schmallenberg Virus

Technical Appendix

Supplementary References

41. Hart TJ, Kohl A, Elliott RM. Role of the NSs protein in the zoonotic capacity of orthobunyaviruses. Zoonoses Public Health. 2009;56:285–96. PubMed http://dx.doi.org/10.1111/j.1863-2378.2008.01166.x

42. ProMED-mail. Schmallenberg virus—Europe (26): vector, morphology. 2012 [cited 2012 May 2]. http:// www.promedmail.org, archive no. 20120311.1066949.

43. Rasmussen LD, Kristensen B, Kirkeby C, Rasmussen TB, Belsham GJ, Bødker R, et al. Culicoids as vectors of Schmallenberg virus. Emerg Infect Dis. 2012;18:1204–6. PubMed http://dx.doi.org/10.3201/eid1807.120385

44. ProMED-Mail. Schmallenberg virus—Europe (35): update, Italy, vector. 2012 [cited 2012 May 2]. http://www.promedmail.org/, archive no. 20120429.1117755.

45. Lo MK, Rota PA. The emergence of Nipah virus, a highly pathogenic paramyxovirus. J Clin Virol. 2008;43:396–400. PubMed http://dx.doi.org/10.1016/j.jcv.2008.08.007

46. Koopmans M, Wilbrink B, Conyn M, Natrop G, van der Nat H, Vennema H, et al. Transmission of H7N7 avian influenza A virus to human beings during a large outbreak in commercial poultry farms in the Netherlands. Lancet. 2004;363:587–93. PubMed http://dx.doi.org/10.1016/S0140-6736(04)15589-X

47. de Wit E, Fouchier RA. Emerging influenza. J Clin Virol. 2008;41:1–6. PubMed http://dx.doi.org/10.1016/j.jcv.2007.10.017

48. Vijgen L, Keyaerts E, Lemey P, Maes P, Van Reeth K, Nauwynck H, et al. Evolutionary history of the closely related group 2 coronaviruses: porcine hemagglutinating encephalomyelitis virus, bovine coronavirus, and human coronavirus OC43. J Virol. 2006;80:7270–4. PubMed http://dx.doi.org/10.1128/JVI.02675-05

49. Mackenzie JS, Williams DT. The zoonotic flaviviruses of southern, south-eastern and eastern Asia, and Australasia: the potential for emergent viruses. Zoonoses Public Health. 2009;56:338–56. PubMed http://dx.doi.org/10.1111/j.1863-2378.2008.01208.x

50. MacDonald G. The epidemiology and control of malaria. London: Oxford University Press; 1957.

51. Elbers ARW, Loeffen WLA, Quak S, de Boer-Luijtze E, van der Spek AN, Bouwstra R, et al. Seroprevalence of Schmallenberg virus antibodies among dairy cattle, the Netherlands, winter 2011–2012. Emerg Infect Dis. 2012;18:1065–71. PubMed http://dx.doi.org/10.3201/eid1807.120323

52. Ducomble T, Wilking H, Stark K, Takla A, Askar M, Schaade L, et al. Lack of evidence for Schmallenberg virus infection in highly exposed persons, Germany, 2012. Emerg Infect Dis. 2012;18:1333–5. PubMed http://dx.doi.org/10.3201/eid1808.120533
